# Efficacy and safety of Chinese medicine JCM-16021 for diarrhea-predominant irritable bowel syndrome: study protocol for a multi-center, randomized, double-blind, placebo controlled clinical trial

**DOI:** 10.1186/s13020-021-00530-2

**Published:** 2021-11-13

**Authors:** Ya Zheng, Jessica Ching, Chung Wah Cheng, Wai Ching Lam, Kam Leung Chan, Xuan Zhang, Pui Yan Lam, Xing Yao Wu, Linda L. D. Zhong, Pei Hua Cao, Cho Wing Lo, Pui Kuan Cheong, Zhixiu Lin, Matthew Koh, Justin Wu, Zhao Xiang Bian

**Affiliations:** 1 Hong Kong Chinese Medicine Clinical Study Centre, School of Chinese Medicine, Hong Kong Baptist University, Hong Kong, SAR China; 2 Hong Kong Institute of Integrative Medicine, The Chinese University of Hong Kong, Hong Kong, China; 3 S. H. Ho Centre for Digestive Health, Institute of Digestive Disease, Faculty of Medicine, The Chinese University of Hong Kong, Hong Kong, SAR China; 4 School of Chinese Medicine, Faculty of Medicine, The Chinese University of Hong Kong, Hong Kong, China; 5 Clinical Research Center, Zhujiang Hospital, Southern Medical University, Guangzhou, China; 6 Department of Surgery, Faculty of Medicine, The Chinese University of Hong Kong, Hong Kong, China

**Keywords:** Irritable bowel syndrome, Diarrhea-predominant, Randomized controlled trial, Chinese medicine JCM-16021, Treatment

## Abstract

**Background:**

Irritable bowel syndrome (IBS) is a common gastrointestinal functional disease. Adults with IBS may experience abdominal pain, change of bowel habits, and abnormal stool form without organic disease. IBS can seriously affect their work productivity and quality of life, especially diarrhea-predominant irritable bowel syndrome (IBS-D). The Chinese medicine JCM-16021 has been shown to be potentially effective in improving the symptoms of IBS-D based on a small scale clinical trial. Hence, a large scale clinical study is designed to further evaluate the efficacy and safety of the Chinese medicine JCM-16021 for IBS-D with traditional Chinese medicine (TCM) pattern of Liver Stagnation and Spleen Deficiency (LSSD).

**Methods:**

This study is a multi-center, randomized, double-blind, placebo-controlled clinical trial. 392 eligible participants will be enrolled with 2-week run-in, 8-week treatment and 8-week follow-up. After run-in period, participants will be randomized to receive either the Chinese medicine JCM-16021 or placebo for 8 weeks, and will have post-treatment follow up for another 8 weeks. The primary outcome is the improvement rate on the global assessment of improvement (GAI) at week 10. The secondary outcomes consist of changes of IBS-D symptoms, TCM pattern improvement, IBS-Quality of Life (IBS-QoL), IBS-Symptom Severity Score (IBS-SSS), safety, etc.

**Results:**

A standard protocol has been developed for the study. The protocol will provided a detailed procedure to conduct a clinical trial and verify if the Chinese medicine JCM-16021 would significantly improve the overall symptoms of IBS-D with LSSD pattern of TCM by relieving abdominal pain, reducing stool frequency, improving the stool consistency and improving quality of life. The consolidated evidence from the study can shed light on the treatment of IBS-D with Chinese medicine.

**Conclusion:**

The protocol will provide details for investigators about the study following SPIRIT Statement. High-quality evidence on the efficacy and safety of Chinese medicine JCM-16021 for IBS-D will be provided through strict compliance with the protocol.

*Trial registration*: ClinicalTrial.gov identifier: NCT03457324. Registered 8 February 2018, https://clinicaltrials.gov/ct2/show/NCT03457324?term=NCT03457324&draw=2&rank=1

**Supplementary Information:**

The online version contains supplementary material available at 10.1186/s13020-021-00530-2.

## Background

Irritable bowel syndrome (IBS) is a common functional gastrointestinal disorder, which is diagnosed on the basis of recurrent abdominal pain related to defecation or in association with a change in stool frequency and form [1]. Furthermore, no anatomical causes have been found 2]. According to a meta-analysis, the pooled estimate of international IBS prevalence is about 11.2% [3]. In Hong Kong, based on Rome II criteria, the prevalence was reported to be 6.6%, of which the proportion of diarrhea-predominant irritable bowel syndrome (IBS-D) was 27% [4].

The pathogenesis of IBS is currently unclear as none of its anatomical causes could be found. It is caused by multiple factors, which include psychological factors, changes in gastrointestinal motility, neural and endocrine factors, infection, diet, drugs etc. [5, 6]. Under the influences of the above factors, the intestinal function is disturbed and that causes abdominal pain, abdominal bloating, constipation or diarrhea. At present, the more accepted mechanisms of IBS include visceral hypersensitivity, abnormal gut motility, abnormal fecal transmission, and mental factors [7]. Recently, some studies show that IBS-D is the most common subtype in IBS based on Rome IV criteria [8–10].

IBS-D greatly affects the quality of life of people. Individuals suffering from IBS-D always complain that they have to avoid traveling or going out because of concerns about toilet access [11]. However, there is still no very satisfactory therapy or medicine for IBS-D yet. According to a survey in America, people’s satisfaction with both over-the-counter (OTC) and prescription treatments for IBS-D are low, with about 20% and 25% of participants being very satisfied with OTC and prescription treatment, respectively [12]. Another study of IBS-D also showed that the overall treatment satisfaction was only approximately 20% [13]. In recent years, more and more people have tried to treat their symptoms with traditional Chinese medicine (TCM), which has been evaluated in many clinical studies [14]. A meta-analysis indicated that Chinese medicine was associated with the improvements of global symptom, IBS-Symptom Severity Score (IBS-SSS), and IBS-D symptoms [15].

According to the TCM theory, the Liver Stagnation and Spleen Deficiency (LSSD) is believed to be the major mechanism of IBS-D. The Liver governs Qi movement, and the Spleen Qi can maintain the transportation of water and nutrient throughout the whole body. Liver Qi stagnation affects the proper operation of the Spleen. When the Spleen is offended by Liver Qi Stagnation, diarrhea will occur. Therefore, the treatment should base on soothing the Liver Qi and strengthening the Spleen Qi. The Chinese medicine JCM-16021 is a new Chinese medicine formula composed of seven herbs: *Rhizoma Atractylodis Macrocephalae* (Baizhu) 20 g, *Radix Paeoniae Lactiflorae* (Baishao) 15 g, *Cortex Magnoliae Officinalis* (Houpo) 10 g, *Semen coicis Lachryma-jobi* (Yiyiren) 20 g, *Polygonaceae* (Huotanmu) 20 g, *Fructus Terminaliae Chebulae*(Hezi) 10 g, *Rhizoma Corydalis Yanhusuo* (Yanhusuo) 15 g. It is developed based on Chinese medicine theories and clinical experience and aims to soothe the Liver Qi and strengthen the Spleen Qi and hence treat IBS-D with LSSD pattern. Our previous small scale study indicated that the Chinese medicine JCM-16021 has a potential therapeutic effect on the relief of IBS-D symptoms compared to holopon and placebo [16]. Further, the Chinese medicine JCM-16021 can dose-dependently attenuate visceral hyperalgesia in neonatal maternal separation (NMS) rats by changing the synthesis and metabolism of 5-HT in the colons [17]. Another study also has indicated that the Chinese medicine JCM-16021 can reduce the colonic enterochromaffin (EC) cell hyperplasia and serotonin (5-HT) availability and upregulate the decreased levels of certain mucosal cytokines, especially the T-cell type 1- (T_h_1-) related cytokines in post-inflammation IBS rats [18]. Based on these studies, we hypothesize that the Chinese medicine JCM-16021 will be an effective remedy for IBS-D. This protocol can provide details for investigators about the study following the Standard Protocol Items for Clinical Trials with Traditional Chinese Medicine 2018 (SPIRIT-TCM Extension 2018), while the final study results will be reported following the CONSORT Extension for Chinese Herbal Medicine Formulas (CONSORT-CHM Formulas 2017) [19–21].

## Objective

The major objectives of this study are as follows: (1) investigate the efficacy of the Chinese medicine JCM-16021 for IBS-D participants with LSSD pattern of TCM; and (2) assess the safety of the Chinese medicine JCM-16021.

## Methods/design

**Fig. 1:**
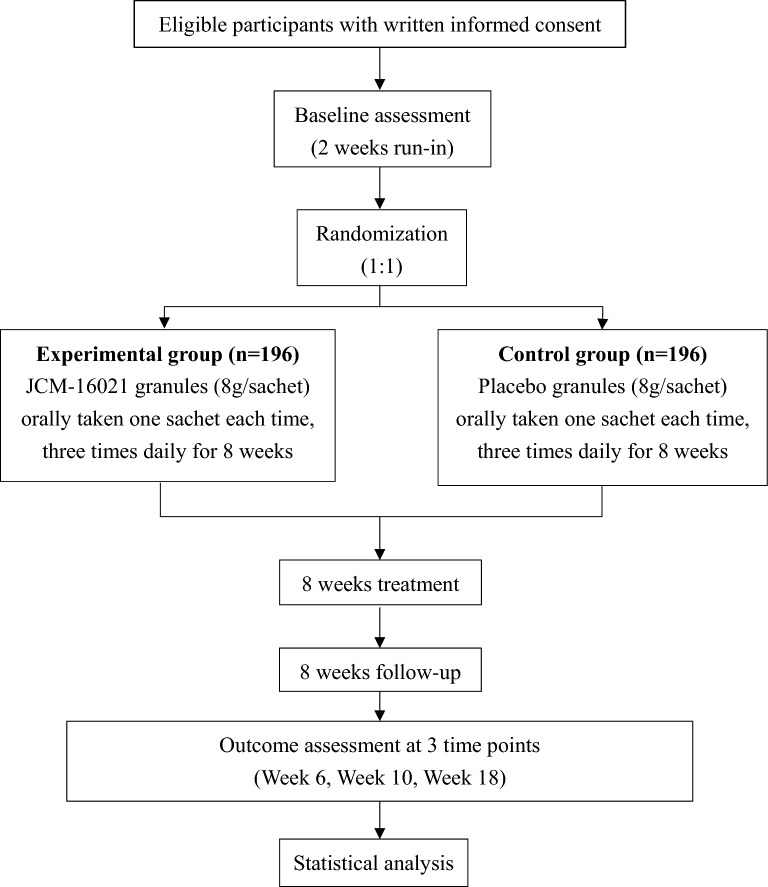
Flow chart

**Fig. 2:**
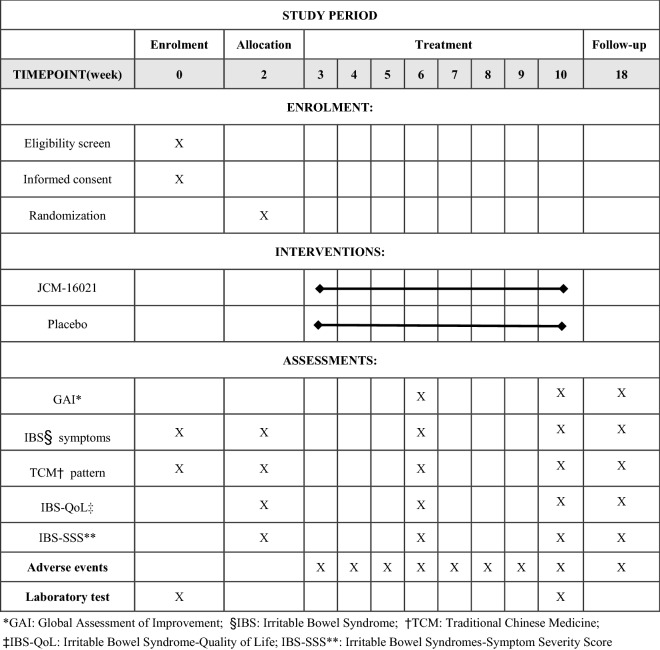
The schedule of enrolment, interventions, and assessments. *GAI: Global Assessment of Improvement; §IBS: Irritable Bowel Syndrome; †TCM: Traditional Chinese Medicine; ‡IBS-QoL: Irritable Bowel Syndrome-Quality of Life; IBS-SSS**: Irritable Bowel Syndrome-Symptom Severity Score

The study is a multi-center, randomized, double-blind, placebo controlled clinical trial. In total, the whole study will last for 18 weeks and schedule five visits, including 2 weeks of the run-in period, 8 weeks of the treatment period, and 8 weeks of the follow-up period. At week 0, the first screening visit will be conducted to include potential participants. Week 0–2 belongs to run-in period. At week 2, the second visit will be conducted to assess the baseline of participants. Then, those eligible participants will be randomized in 1:1 ratio to receive either the Chinese medicine JCM-16021 or placebo. At week 6, the third visit will be conducted to assess the situation after treatment of 4 weeks. At week 10, the fourth visit will be conducted to assess the situation after treatment of 8 weeks. Then, these participants will be followed up with another 8-week to assess the situation after stopping the medicine and the fifth visit will be conducted at week 18. Participants will be assessed on the scheduled day for each visit by face to face at the designated clinics. The flowchart and schedule of the trial are shown in Figs. 1 and 2, including schedule of enrolment, interventions and assessments. The trial protocol is written in accordance with SPIRIT-TCM Extension 2018 checklist [14] (Additional file 1). And the structured study summary is described in Table 1 according to the World Health Organization Trial Registration Data Set.

**Table 1:** WHO trial registration data set—structured summary

Data category	Information
Primary registry, trial identifying number	ClinicalTrials.gov (NCT03457324)
Date of registration in primary registry	February 8, 2018
Secondary identifying numbers	
Sources of monetary support	Innovative Technology Commission of the government of Hong Kong Special Administrative Region
Contact for public queries	ZXB, MD, PHD [bzxiang@hkbu.edu.hk]
Contact for scientific queries	ZXB, MD, PHD [bzxiang@hkbu.edu.hk]
Public title	Efficacy and Safety of Chinese Medicine JCM-16021 for Diarrhea-predominant Irritable Bowel Syndrome
Scientific title	Efficacy and Safety of Chinese Medicine JCM-16021 for Diarrhea-predominant Irritable Bowel Syndrome: Study protocol for a Multi-center, Randomized, Double-blind, Placebo Controlled Clinical Trial
Country of recruitment	Hong Kong, China
Health problem	Diarrhea-predominant Irritable Bowel Syndrome
Intervention(s)	Experimental group: JCM-16021 granules (8 g/sachet for each time, three times daily for 8 weeks)
	Control group: Placebo granules (8 g/sachet for each time, three times daily for 8 weeks)
Key inclusion and exclusion criteria	Inclusion criteria: (1) the diagnosis criteria of IBS-D; (2) the diagnosis of LSSD; (3) Age of 18–65 years; (4) with written informed consent
	Exclusion criteria: (1) Constipation predominant, mixed or unsubtyped IBS; (2) Abnormal colonoscopy within 5 years (except for benign polypectomy or hemorrhoids); (3) With severe diseases on the system of heart, lung, liver, and kidney which are diagnosed by TCM theory; (4) Concomitant diabetes, Glucose-6-Phosphate Dehydrogenase deficiency, unstable hypertension, malignant tumor or have undergone thyroid surgery or medication within a year; (5) With diseases on the spleen-stomach system or other diseases that affect the movement of qi on the spleen-stomach system, which are diagnosed by TCM theory; (6) Current concomitant medication with effects on gastrointestinal function (e.g. anticholinergic drugs, calcium channel blockers, 5-HT3 receptor antagonists, antidiarrheal agents, antacids, prokinetic agents, antidepressants, anxiolytics and intestinal flora regulating drugs); (7) Medical history of gastrointestinal surgery (except for appendicitis surgery); (8) The serum levels of alanine transaminase (ALT), aspartate transaminase(AST), and creatinine(Cr) exceed 1.5 times of the reference limit; (9) History of allergy in Chinese medicine; (10) Women in pregnancy or breast-feeding; (11) Medical history of neurological diseases or psychiatric disorders; (12) Currently participating in another clinical trial; (13) Taking IBS treatment drugs within 1 week
Study type	Interventional allocation: randomized
	Intervention model: parallel assignment
	Masking: double blind (participants and Physicians, Chinese Medicine Practitioners and research assistants)
	Primary purpose: Treatment
	Phase III
Date of first enrollment	October 2018
Target sample size	392
Recruitment status	Completed
Primary outcome	The improvement rate on the Global Assessment of Improvement (GAI) at week 10
Key secondary outcomes	Changes of IBS symptoms, TCM pattern improvement, IBS-Quality of Life (IBS-QoL), IBS-Symptom Severity Score (IBS-SSS), safety

### Setting of the study

The study will be simultaneously conducted in three centers, namely Hong Kong Baptist University Mr. and Mrs. Chan Hon Yin Chinese Medicine Specialty Clinic and Good Clinical Practice Centre, Hong Kong Baptist University HK Island Wei Ke Qiang Chinese Medicine Specialty Clinic, and Center for Integrative Medicine, Hong Kong Institute of Integrative Medicine, The Chinese University of Hong Kong.

### Randomization

The randomization sequence will be generated using block randomization with a fixed block size by our statistician with SPSS statistical analysis software. The sequence will be kept in opaque sealed envelopes with consecutive numbers. These envelopes will be prepared by pharmaceutical company. The envelopes numbered 001–100 and 201–392 will be kept at the site of Hong Kong Baptist University. The envelopes numbered 101–200 will be kept at the site of the Chinese University of Hong Kong. Chinese medicine practitioners responsible for the recruitment and evaluation will be blinded. Each center will have a research assistant responsible for allocating drugs to participants. The research assistant will open the same numbered envelope based on the randomization enrolment number when an eligible participant can be included in the trial. Then, the research assistant will assign the drugs to the participant according to the sequence. The eligible participants will be randomized and assigned to the Chinese medicine JCM-16021 group or the placebo group in 1:1 ratio (196 cases/group).

### Blinding

The trial is a double-blinded design, in which all the Chinese medicine practitioners, research assistants and participants are ignorant of the treatment assignments. The physical appearance of placebo is consistent with the Chinese medicine JCM-16021, including the packaging, appearance, color and taste. Pharmaceutical company will be responsible for labelling the Chinese medicine JCM-16021 and placebo according to the blinding codes. ‘16021A’ and ‘16021B’ will be used to label the interventions. The blinding code will be kept strictly confidential by the principal investigators until completion of the final statistical analysis and report of the trial. Only if there is a serious adverse event (SAE) relevant to the research medication, the assignment code will be broken. The date and reason for breaking the blinding code should be recorded in the case report form (CRF). Relevant institutions, including the Research Ethics Committee of Hong Kong Baptist University and Department of Health of Hong Kong Special Administrative Region should be informed accordingly within 24 h.

### Recruitment

The recruitment of the study is via the press conferences, announcements on university website, advertisements on local newspapers or Facebook, promotion posters and leaflets, and specialist’s referral. People who are interested in the trial can contact the researchers by telephone or e-mail. Researchers will conduct preliminary screening on the phone and invite these potential candidates to come to the clinics to have a further screening face to face. Chinese medicine practitioners will be responsible for conducting the screening and explaining the details of the study, such as content, arrangement, and possible risks etc. The eligible participants will be invited to join the study after signing the informed consent form.

### Inclusion and exclusion criteria

According to Rome IV Criteria, IBS is diagnosed based on recurrent abdominal pain, on average, at least one day per week in the last 3 months and associated with any two or more of followings. They are related to defecation, change in frequency of stool, or change in form (appearance) of stool. Meanwhile, diarrhea predominant is classified by loose (mushy) or watery stools > 25% and hard or lumpy stool < 25% of bowel movements [22].

The diagnostic criteria of LSSD pattern of TCM is in reference to the “Guiding Principles for Clinical Study of New Chinese Medicines” [23]. LSSD is diagnosed when all the main symptoms and signs and associating with two or more secondary symptoms and signs are fulfilled. The main symptoms and signs include abdominal pain and diarrhea (inducing or aggravating by emotional upset or stress), and changes with defecation. Secondary symptoms and signs include borborygmus and passing gas frequently, distention and fullness in chest and hypochondrium or frequent sighing, emotional depression or irritability, anorexia and abdominal bloating, and pale red tongue with white thin fur and string like pulse.

Participants age of 18–65 years will be included if they fulfill of the diagnosis criteria of IBS-D based on Rome IV criteria, and the diagnosis of LSSD pattern of TCM based on “Guiding Principles for Clinical Study of New Chinese Medicines”, together with written informed consent.

Participants will be excluded if they are constipation predominant, mixed or unsubtyped IBS, abnormal colonoscopy within 5 years (except for benign polypectomy or hemorrhoids), with severe diseases on the system of heart, lung, liver, and kidney which are diagnosed by TCM theory, concomitant diabetes, Glucose-6-Phosphate Dehydrogenase deficiency, unstable hypertension, malignant tumor or have undergone thyroid surgery or medication within a year, and with diseases on the Spleen-Stomach system or other diseases that affect the movement of Qi on the Spleen-Stomach system, which are diagnosed by TCM theory. Participants with current concomitant medication with effects on gastrointestinal function (e.g., anticholinergic drugs, calcium channel blockers, 5-HT3 receptor antagonists, antidiarrheal agents, antacids, prokinetic agents, antidepressants, anxiolytics and intestinal flora regulating drugs), medical history of gastrointestinal surgery (except for appendicitis surgery), serum levels of alanine transaminase (ALT), aspartate transaminase (AST), and creatinine (Cr) exceed 1.5 times of the reference limit, history of allergy in Chinese medicine, and medical history of neurological diseases or psychiatric disorders will also be excluded. Women in pregnancy or breast-feeding, or subjects currently participating in another clinical trial or taking IBS treatment drugs within 1 week are not eligible to be enrolled in this study.

During the study, participants may opt to withdraw at any time. Those who are lost to follow up or withdraw from the study after randomization will be treated as drop-out cases.

### Intervention

The Chinese medicine JCM-16021 is composed of seven herbs, namely*Rhizoma Atractylodis Macrocephalae* (Baizhu) 20 g, *Radix Paeoniae Lactiflorae* (Baishao) 15 g, *Cortex Magnoliae Officinalis* (Houpo) 10 g, *Semen coicis Lachryma-jobi* (Yiyiren) 20 g, *Polygonaceae* (Huotanmu) 20 g, *Fructus Terminaliae Chebulae* (Hezi) 10 g, *Rhizoma Corydalis Yanhusuo* (Yanhusuo) 15 g. These herbs are processed in granules form and will be packaged in a sachet of 8 g with aluminum foil composite film. Every 21 sachets will be packed in a transparent bag with label for 1-week course. The placebo is made from caramel (0.55%), sunset yellow (0.01%), tartrazine (0.06%), gardenia yellow (0.06%), aspartame (0.8%), sucrose octaacetate (0.02%), starch (49.25%), and dextrin (49.25%). The form, color, package, usage, and dosage of placebo is consistent with the Chinese medicine JCM-16021 granules, as is shown in Additional file 2. Participants will take either the Chinese medicine JCM-16021 granules or placebo granules (8 g/sachet) orally, one sachet each time, three times daily half an hour after a meal for 8 weeks.

All investigational medicinal product (IMP) including the Chinese medicine JCM-16021 granules and placebo are produced by the Purapharm (Nanning) Pharmaceutical Co. Ltd. The entire manufacturing process will be in strict compliance with the standards of Good Manufactory Practice (GMP). The production process of the Chinese medicine JCM-16021 granules and placebo granules will be compliant with the quality specification standards, respectively. According to the Chinese Pharmacopoeia (2015), all qualified IMP will be delivered to the site of clinical study and stored within a range of 25 ± 2 °C and relative humidity range of 60 ± 10% [24]. The investigators are responsible for ensuring adequate accountability of all used and unused IMP. For better guaranteeing the safety and quality of the Chinese medicine JCM-16021 granules, accelerated stability tests, heavy mental and toxic elements, pesticides residues, and microbial limit will be conducted.

### Primary outcome

The primary outcome is the improvement rate on the global assessment of improvement (GAI) score at week 10, same as the previous small scale study [16]. The participants will be asked to give a global assessment of their IBS symptoms at visit 3, 4 and 5 with the question “In the past 7 days, compared with your IBS symptoms before you started the trial, are you now: substantially worse, moderately worse, slightly worse, no change, slightly improved, moderately improved, or substantially improved? [25]’’ The global symptom improvement will be evaluated by scales (substantially worse = 0, moderately worse = 1, slightly worse = 2, no change = 3, slightly improved = 4, moderately improved = 5, substantially improved = 6). The 0–2 score, 3 score and 4–6 score represent worsen, no change and improvement, respectively. Improvement rate of GAI is defined as the proportion of participants with improvement (in scores of 4 or greater).

### Secondary outcomes

#### IBS symptoms

IBS symptoms, including abdominal pain (with scores from 0 to 10 representing none to most severe), stool frequency and stool consistency will be recorded by participants in a diary throughout 18 weeks of study. Investigators will grade each symptom by cardinal symptoms evaluation quantitation scale (None = 0, Mild = 1, Moderate = 2, Severe = 3) shown in Additional file 3: Table S1. The changes of these IBS symptoms before and after the treatment will be used for efficacy evaluation. The details are as below:

i. Pain responder rate in daily worst abdominal pain scores: According to the guidance of the U.S. FDA, pain responder is defined as that participant who meets the daily pain response criteria for at least 50% of the days with diary entries during the observational period of interest. Daily pain response is defined as the ≥ 30% decrease in the worst abdominal pain scores in the past 24 h compared to baseline (average of daily worst abdominal pain the 2-week prior to randomization). Pain score is ranked from 0 to 10 representing none to the most severe [26].
ii. Stool consistency responder rate in daily stool consistency scores: Stool consistency responder is defined as that participant who meets daily stool consistency response criterion (i.e., score of 1, 2, 3, or 4 or absence of bowel movement if accompanied by ≥ 30% decrease in worst abdominal pain scores compared to baseline pain) for at least 50% of days with diary entries during the observational period of interest. Bristol stool scale is defined as 7-point Scale in which a score of 1 = separate hard lumps, 2 = sausage shaped but lumpy, 3 = sausage-like with cracks on the surface, 4 = sausage-like but smooth and soft, 5 = soft blobs with clear cut edges, 6 = fluffy pieces with ragged edges, and 7 = watery with no solid pieces [26].
iii. Improvement rate and efficacy rate*:* The standards of efficacy assessment of single symptom are as follows: (a) Excellent: symptoms disappearing; (b) Effective: symptom score decreased ≥ 2 points; (c) Helpful: symptom score decreased 1 point; (d) Invalid: no change in symptom score. Improvement rate is defined as the proportion of participants in excellent, effective and helpful. Comprehensive efficacy judgement standard of cardinal symptoms is calculated by “(total scoring of prior treatment − total scoring of post treatment)/total scoring of prior treatment × 100%”. Clinical remission is defined as symptom disappearing, symptom improvement (≥ 80%), helpful (between 50 and 80%), invalid (< 50%). Total effective rate is defined as the proportion of participants with clinical remission and symptom improvement [23].

#### TCM pattern

Five typical symptoms for LSSD are assessed to evaluate the changes of TCM Pattern, involving: (i) abdominal distension, (ii) borborygmus and flatus, (iii) distension and fullness in chest and hypochondrium, (iv) frequent sighing, poor appetite, and (v) mental depression or irritability. Investigators will grade the TCM pattern scale (None = 0, Mild = 1, Moderate = 2, Severe = 3) as shown in Additional file 3: Table S2. Efficacy assessment standards of Syndrome of Chinese medicine are as follows: Clinical remission: clinical symptoms and signs disappear or basically disappear, total scoring declining ≥ 95%; Excellence: clinical symptoms and signs are significantly improved, total scoring declining ≥ 70%; Effective: clinical symptoms and signs are improved, total scoring declining ≥ 30%; Invalid: clinical symptoms and signs are without obvious improvement or even with exacerbation, total scoring declining < 30%. The effective rate on TCM pattern will be evaluated with following calculation formula (nimodipine method): [(total score of prior treatment − total score of post treatment)/total score of prior treatment] × 100% [23].

#### Irritable bowel syndrome-quality of life

The health-related quality of life of participants will be measured with validated Irritable Bowel Syndrome-Quality of Life (IBS-QoL) questionnaire, which includes 34 items and involves eight aspects: dysphoria (Q1), interference with activity (Q2), body image (Q3), health worry (Q4), food avoidance (Q5), social reaction (Q6), sexual (Q7) and relationship (Q8) [27]. Each item will be rated on a 5-point Likert scale. Higher score reflects better quality of life. The difference of score before and after the medication will be evaluated.

#### Irritable bowel syndrome-symptom severity score

The IBS-SSS questionnaire will be completed at visit 2, 3, 4, 5. The IBS-SSS involves five aspects that measures on the severity of abdominal pain, the frequency of abdominal pain, the severity of abdominal discomfort, defecation satisfaction, and interference with daily life. The range of score is 0–500 points scale [28]. The change of score before and after the medication will be evaluated.

### Safety outcome

In this study, adverse events (AEs) or SAEs related to IMPs are used for safety evaluation, including new incurrence symptoms or diseases, abnormal vital signs, clinically significant abnormal laboratory examination on renal or liver function. The severity of AEs is graded based on the Common Terminology Criteria for Adverse Events v4.0.

### Quality control and assurance

Firstly, all investigators will be required to complete the TRREE on-line training programme on the ethics and regulation of health research involving human participants (https://elearning.trree.org/) and get the online certification of Good Clinical Practice (GCP) before the start of the study. Secondly, all investigators will study the details of the protocol and investigator’s brochure together at the research centre of Hong Kong Baptist University. If there are any modifications, investigator will also study the revision of the protocol and the investigator’s brochure in time. Thirdly, a regular monthly meeting will be held to report the study progress to principal investigators (i.e., recruitment, compliance, adverse events, etc.), assess the investigators’ activities (i.e., data collection, data entry, compliance with the protocol and investigator’s brochure, etc.), discuss and solve the problems (if any). In general, the meeting will be conducted at the office of Hong Kong Baptist University.

In addition, investigators are required to explain details of clinical trial to participants and obtain informed consent. Investigators should also let participants to be fully informed of the significance of trial and importance of medication. For participants with low compliance, investigators should follow the participant in time and record the reasons in details.

### Data collection, management and monitoring

All data will be collected through patient diaries, questionnaires, and CRFs. All enrolled participants have to record their abdominal pain, stool frequency, stool consistency in a paper-based patient diary throughout 18 weeks of study. At each visit, participants will be required to return the diaries and complete some questionnaires, including the GAI questionnaire, the IBS-QoL questionnaire, and the IBS-SSS questionnaire. Chinese medicine practitioners and study coordinators have to fill in the CRFs to record relevant information, such as blood pressure, the changes of TCM Pattern, and compliance/noncompliance with research medication and concomitant conventional maintenance therapy, etc. These data collected in visit 2 will be the baseline data. All personal information of participants will be kept confidential. The Research Electronic Data Capture (REDCap) [29, 30] will be used to collect and manage all participants’ data by researchers. All centers have corresponding personnel to input the data timely. The researchers with authorized accounts can log in and browse the data. All raw data collected by patient diaries, questionnaires, and CRFs could be shared based on the request and special approval from the team. During the whole study, all AEs (new incurrence symptoms or diseases, abnormal vital signs, clinically significant abnormal laboratory examination) should be recorded in CRF and investigators are also required to fill in the relevant information in the “Adverse Event Report Form”, which includes start time, duration, severity, and relationship with IMP etc. Once there is an AE which results in hypersensitivity towards research medication or significant abnormality on liver or renal function, investigators can suspend or terminate the IMP, and report to principal investigators in time. If there are any SAEs, investigators should fill in the “Serious Adverse Event Report Form” with signature and report to the Research Ethics Committee within 24 h. Also, all AEs and SAEs are required to be reported to the Department of Health of Hong Kong Special Administrative Region every year. The trial document will be kept for 7 years after the completion of the trial. The Research Ethics Committee of Hong Kong Baptist University and the Department of Health of Hong Kong Special Administrative Region play a role to monitor the data. The principal investigators need to submit the progress reports to the Research Ethics Committee of Hong Kong Baptist University and the Department of Health of Hong Kong Special Administrative Region every year, and to the funding agency Innovative Technology Commission of the Government of Hong Kong Special Administrative Region at every 6 months.

### Biological specimen collections

During the clinical study, the blood, urine and feces will be collected from all participants with written informed consent on 2nd and 10th week, respectively. All samples will be delivered to the laboratory of the School of Chinese Medicine, Hong Kong Baptist University and be stored at − 80 °C for the metabolomics and metagenomics sequencing analysis. Blood, urine and fecal samples will be collected to analyze the metabolites profile of gut microbiota and the host before and after the treatment. Metagenomics sequencing will be used to monitor the gut microbiota composition profile before and after treatment. Both metabolomics and metagenomics sequencing are served as the methods to explore the mechanism of the Chinese medicine JCM-16021 attenuating the bowel symptoms by modulating gut microbiota.

### Sample size calculation

From our previous study, improvement rate on the GAI was 52% in the Chinese medicine JCM-16021 group and 32% in western medicine group, respectively [18]. According to the previous clinical trial guidelines for pharmacological treatment of IBS, a 15% improvement of the global outcome measure over placebo could be considered as a clinically significant therapeutic gain [31]. We assumed that there would be a 15% difference in the proportion of participants who report an improvement in their global symptoms after 8 weeks of treatment (either slight, moderate, or substantial) between the placebo group and the Chinese medicine JCM-16021 group. Thus, 166 participants are required in each group with 80% power and two-sided 5% alpha. Further assuming a 15% drop-out rate, a total of 392 participants (196 per arm) will be enrolled to ensure that the study is sufficiently powered to answer the research question of interest.

### Statistical analysis

Baseline data will be descriptively summarized. Categorical variables will be summarized by frequencies and percentages. Continuous variables will be summarized with mean and standard deviation [mean ± SD].

For the outcome analysis, comparisons between two groups for continuous variables will be conducted by using an analysis of variance (ANOVA), with other confounding factors like a multicenter character conducting the covariate analysis. Statistical analysis for the data which do not meet above conditions (e.g., non-normal) will be conducted with the use of non-parametric test. Categorical data of different groups will be reported as frequency (proportion). Comparisons between groups for these categorical data will be assessed with the use of chi-square test (CMH test) or non-parametric testing. For the repeatedly measured outcomes, the difference with the baseline data (follow-up minus the baseline) will be analyzed and the baseline data will be included in the model as covariables. Subgroup analyses will be carried out irrespective of whether there is a significant treatment effect on the primary outcome. The participants’ drop-out will be descriptively summarized. Both per-protocol analysis (PP) and intention-to-treat (ITT) analysis will be used to conduct the efficacy analysis. All treatment groups’ comparisons except for the primary outcome will be performed at a two-sided level of 0.05.

The safety analysis will mainly be performed by analysis of the incidence of adverse events between groups and the incidence of abnormal laboratory data before and after treatment between groups. A detailed description of the statistical analysis plan (SAP) is provided in Additional file 3.

## Discussion

Irritable Bowel Syndrome (IBS) is a persistent or intermittent episode of functional gastrointestinal disease with abdominal pain, bowel habits and/or stool traits that change clinically [32]. Many people with IBS are eager for an effective treatment. However, at present, due to the complexity and uncertainty of its etiology, there is no clear biochemical diagnostic index and current treatment is not very satisfactory. Therefore, it is important to investigate an effective medicine for IBS. Many RCTs have suggested that TCM formulas have significant efficacy for IBS symptoms. The mechanisms of these TCM formulas are likely related to the regulation of neurotransmitters and hormones in the enteric nervous system, attenuation of intestinal inflammation and restoration of intestinal flora, etc. [33, 34]. JCM-16021 is a new Chinese medicine formula developed by our team for the treatment of IBS-D based on the Chinese medicine theories and clinical experience. Our research group has conducted a series of studies on the Chinese medicine JCM-16021 to evaluate its pharmacology, efficacy, safety for more than 10 years. In the previous small scale clinical trial, it has shown a trend that participants with the Chinese medicine JCM-16021 had the greatest extent of improvement at the end of 8-week treatment and the end of 8-week follow up [16]. But a large scale trial is needed to further evaluate the efficacy and safety of this formula for IBS-D. Recently, disorder of the gut microbiota has been considered to be one of potential aetiological factor [32, 35–37]. Our previous basic studies have also shown that the Chinese medicine JCM-16021 could effectively relieve intestinal hyperalgesia by altering the gut microbiota and improving the co-metabolism of the gut microbiota and its host, and also through regulating host’s immune response to gut microbiota as well as enhancing gut barrier. It is likely to be an important mechanism for the effective treatment of IBS-D with the Chinese medicine JCM-16021[38]. This study will provide an opportunity to reassess the mechanism in a large scale trial.

## Conclusion

In conclusion, this protocol will provide details for investigators about the study following SPIRIT Statement for Chinese Herbal Medicine Formulas. This study has a larger sample size than our previous pilot study. It is believed that more higher-quality evidence on the efficacy and safety of the Chinese medicine JCM-16021 for IBS-D will be provided through strictly compliance with the protocol. The results may also help to discover the underlying mechanism of this herbal remedy for the condition.

## Trial status

Participants’ recruitment started in October 2018. Today, 392 participants have been recruited. The actual date for the start of recruitment was the 8th November 2018. The actual date for the end of recruitment was the 29th October 2020. The last participant completed the follow-up on 17th February 2021.

## Supplementary Information

**Additional file 1. **SPIRIT-TCM Extension 2018 checklist.

**Additional file 2.** The form, color, package, and lable of investigational medicinal products.

**Additional file 3. **Statistical Analysis Plan.

**Additional file 4. **Informed Consent Form.

## Data Availability

The datasets will be available from the corresponding author on reasonable request.

## Abbreviations

IBS: Irritable bowel syndrome
TCM: Traditional Chinese medicine
IBS-SSS: Irritable bowel syndrome-symptom severity score
RCTs: Randomized clinical trials
LSSD: Liver stagnation and spleen deficiency
NMS: Neonatal maternal separation
EC: Enterochromaffin
5-HT: Serotonin
T_h_1-: T-cell type 1-
SPIRIT-TCM Extension 2018: Standard protocol items for clinical trials with traditional Chinese medicine 2018
CONSORT-CHM Formulas 2017: CONSORT extension for Chinese herbal medicine formulas 2017
SAEs: Serious adverse events
CRF: Case report form
ALT: Alanine transaminase
AST: Aspartate transaminase
Cr: Creatinine
IMP: Investigational medicinal product
GMP: Good manufactory practice
GAI: Global assessment of improvement
IBS-QoL: Irritable bowel syndrome-quality of life
AEs: Adverse events
GCP: Good Clinical Practice
CMH: Cochran-Mantel–Haenszel
ANCOVA: Analysis of covariance
PP: Per-protocol analysis
ITT: Intention-to treat analysis
REDCap: Research electronic data capture
SAP: Statistical analysis plan
