# Supplementary material for: Efficacy and safety of Chinese medicine JCM-16021 for diarrhea-predominant irritable bowel syndrome: study protocol for a multi-center, randomized, double-blind, placebo controlled clinical trial
Source: Chin Med. 2021 Nov 13;16:117. doi: 10.1186/s13020-021-00530-2 (PMC8590321; doi:10.1186/s13020-021-00530-2)
Supplement: Supplementary file 2 — Additional file 2. The form, color, package, and lable of investigational medicinal products. [file 13020_2021_530_MOESM2_ESM.pdf]

The form, color, package, and lable of investigational manufactured products

<table>
<tr><th>Drug</th><th>Lable</th><th>Form
Color
Package</th></tr>
<tr><td>16021A</td><td>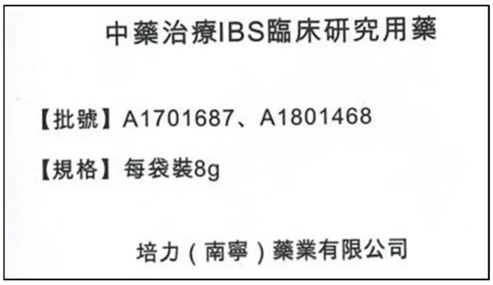
中藥治療IBS臨床研究用藥
【批號】A1701687、A1801468
【規格】每袋裝8g
培力（南寧）藥業有限公司
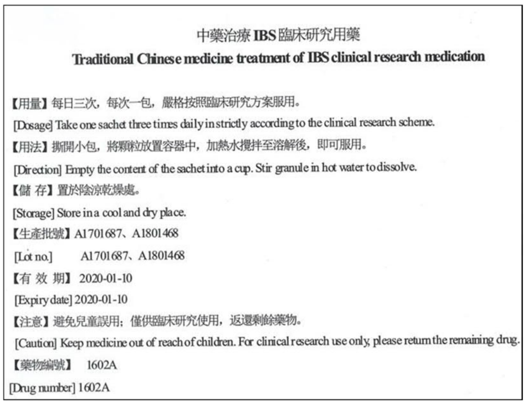
中藥治療 IBS 臨床研究用藥
Traditional Chinese medicine treatment of IBS clinical research medication
【用量】每日三次，每次一包，嚴格按照臨床研究方案服用。
[Dosage] Take one sachet three times daily in strictly according to the clinical research scheme.
【用法】撕開小包，將顆粒放置容器中，加熱水攪拌至溶解後，即可服用。
[Direction] Empty the content of the sachet into a cup. Stir granule in hot water to dissolve.
【儲 存】置於陰涼乾燥處。
[Storage] Store in a cool and dry place.
【生產批號】A1701687、A1801468
[Lot no.] A1701687、A1801468
【有 效 期】2020-01-10
[Expiry date] 2020-01-10
【注意】避免兒童誤用；僅供臨床研究使用，返還剩餘藥物。
[Caution] Keep medicine out of reach of children. For clinical research use only, please return the remaining drug.
【藥物編號】 1602A
[Drug number] 1602A</td><td>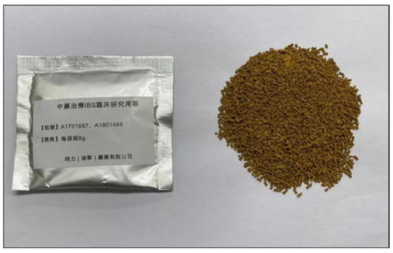
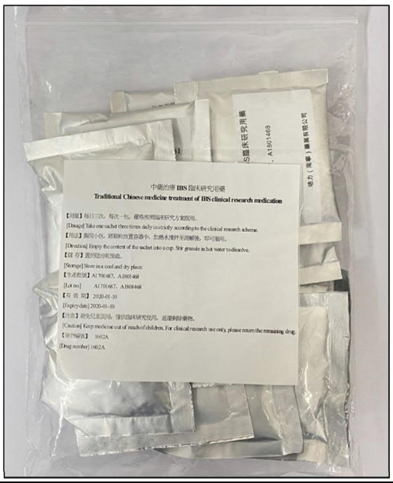</td></tr>
<tr><td>16021B</td><td>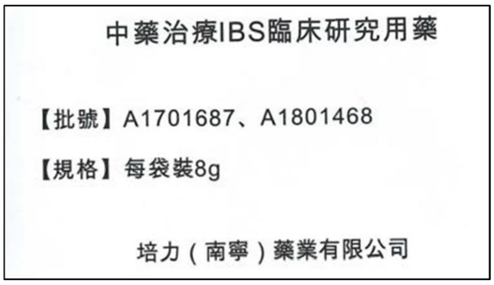
中藥治療IBS臨床研究用藥
【批號】A1701687、A1801468
【規格】每袋裝8g
培力（南寧）藥業有限公司
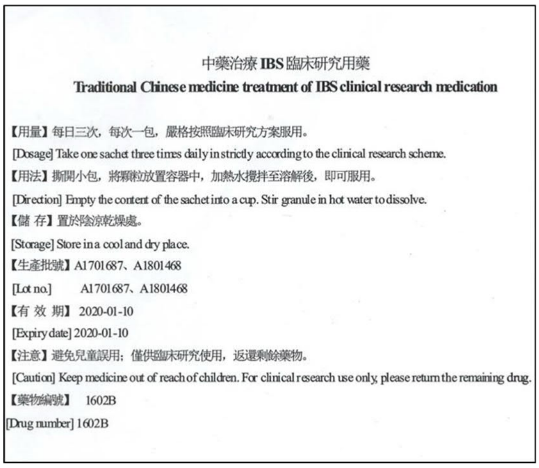
中藥治療 IBS 臨床研究用藥
Traditional Chinese medicine treatment of IBS clinical research medication
【用量】每日三次，每次一包，嚴格按照臨床研究方案服用。
[Dosage] Take one sachet three times daily in strictly according to the clinical research scheme.
【用法】撕開小包，將顆粒放置容器中，加熱水攪拌至溶解後，即可服用。
[Direction] Empty the content of the sachet into a cup. Stir granule in hot water to dissolve.
【儲 存】置於陰涼乾燥處。
[Storage] Store in a cool and dry place.
【生產批號】A1701687、A1801468
[Lot no.] A1701687、A1801468
【有 效 期】2020-01-10
[Expiry date] 2020-01-10
【注意】避免兒童誤用；僅供臨床研究使用，返還剩餘藥物。
[Caution] Keep medicine out of reach of children. For clinical research use only, please return the remaining drug.
【藥物編號】 1602B
[Drug number] 1602B</td><td>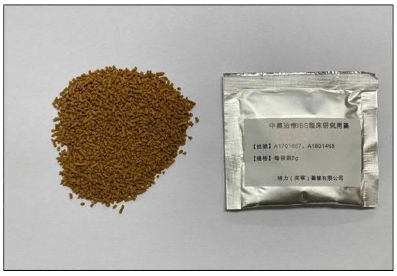
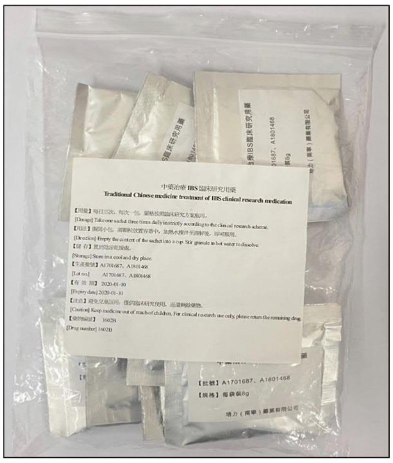</td></tr>
</table>
