# Supplementary material for: Efficacy and safety of Chinese medicine JCM-16021 for diarrhea-predominant irritable bowel syndrome: study protocol for a multi-center, randomized, double-blind, placebo controlled clinical trial
Source: Chin Med. 2021 Nov 13;16:117. doi: 10.1186/s13020-021-00530-2 (PMC8590321; doi:10.1186/s13020-021-00530-2)
Supplement: Supplementary file 3 — Additional file 3. Statistical Analysis Plan. [file 13020_2021_530_MOESM3_ESM.pdf]

Efficacy and Safety of Chinese Medicine JCM-16021 for Diarrhea-predominant Irritable Bowel Syndrome: Study protocol for a Multi-center, Randomized, Double-blind, Placebo Controlled Clinical Trial

STATISTICAL ANALYSIS PLAN

Authors: Ya Zheng, Jessica Ching, Chung Wah Cheng, Wai Ching Lam, Kam Leung Chan, Xuan Zhang, Pui Yan Lam, Xing Yao Wu, Linda L.D.Zhong, Pei Hua Cao, Cho Wing Lo, Pui Kuan Cheong, Zhixiu Lin, Matthew Koh, Justin Wu, Zhao Xiang Bian

Version: 1.0

Contents

Abstract 4

1. Background 5

2. Study design 5

2.1 Overview...5

2.2 Sample size estimation 5

2.3 Definitions of the outcomes...5

2.3.1 Primary outcome 6

2.3.2 Secondary outcomes 6

2.3.3 Safety outcome...7

3. Statistical Analysis 7

3.1 Analysis principles 7

3.2 Trial profile 8

3.3 Patients' characteristics and baseline comparisons.....................................8

3.4 Primary outcome 8

3.5 Secondary outcome 9

3.6 Adverse events..10

3.7 Patients drop out...10

3.8 Subgroup analysis 10

3.9 Sensitivity analysis 10

4. Trial status 10

5. Tables and figures for the main results paper 11

6. References 11

7. Appendix 1 13

8. Appendix 2 20

9. Appendix 3 21

Primary Rationale for Amendment:

There was no amendment for the statistical analysis plan in this study.

List of abbreviations and definition of terms

IBS-D	Diarrhea-predominant Irritable bowel syndrome
TCM	Traditional Chinese Medicine
LSSD	Liver Stagnation and Spleen deficiency
SAP	Statistical Analysis Plan
GAI	Global Assessment of Improvement
CRFs	Case Report Forms
IBS-QoL	Irritable Bowel Syndrome-Quality of Life
AEs	Adverse Events
SAEs	Serious Adverse Events
LOCF	Last-observation-carried forward
PP	Per-protocol analysis
ITT	Intention-to treat
CMH	Cochran-Mantel-Haenszel
CONSORT	Consolidated Standards of Reporting Trials
Mean±SD	Mean and standard deviation
IQR	Interquartile range
CMH	Cochran-Mantel-Haenszel
ANOVA	Analysis of variance

Abstract

Background: Irritable bowel syndrome is a common gastrointestinal functional disease. Patients may experience abdominal pain, change of bowel habits, and abnormal stool form without organic disease. IBS can seriously affect patients' work productivity and quality of life. Chinese Herbal Medicine JCM-16021 has been shown to be a potential effective remedy on the improvement of symptoms of IBS based on a small scale clinical trial. Hence, a large scale clinical study is designed to further evaluate the efficacy and safety of JCM-16021 for diarrhea-predominant Irritable bowel syndrome (IBS-D) with traditional Chinese medicine (TCM) pattern of Liver Stagnation and Spleen Deficiency (LSSD).

Objective: The study is designed to assess the efficacy and safety of JCM-16021 for IBS-D patients with TCM pattern of LSSD.

Methods: This study is a multi-center, randomized, double-blind, placebo controlled clinical trial. All data collected by participating researchers will be reviewed and assessed. Information pertaining to the baseline characteristics of patients will be selected and for each item statistically relevant descriptive elements are described. Information relevant to the JCM-16021 and placebo groups is classified and, for each item, descriptive statistical analyses are planned for comparison between the JCM-16021 and placebo groups. For the outcomes which are classified as primary and secondary, the most appropriate statistical comparison to be made between groups are described.

Results: A statistical analysis plan (SAP) has been developed for the results of our study. This plan will allow a comprehensive description of baseline characteristics, features of the treatments, along with pre-determined statistical assessment of relevant outcome in a way that is transparent, available to the public, verifiable and pre-determined before completion of data collection.

Conclusions: We have developed a pre-determined SAP for the study which is to be followed to avoid analysis bias arising from prior knowledge of the study findings.

Trial registration: ClinicalTrial.gov identifier: NCT03457324. Registered on 8 February 2018.

Keywords: Statistical analysis plan, Irritable bowel syndrome, Diarrhea-predominant, Randomized controlled trial, JCM-16021, Chinese Medicine, and Treatment

1. Background

Irritable bowel syndrome (IBS) is a common gastrointestinal functional disease. Patients may experience abdominal pain, change of bowel habits, and abnormal stool form without organic disease[1, 2]. There is a relative high prevalence for IBS in the world and it can seriously affect patients' work productivity and quality of life and cause a serious socioeconomic burden[3, 4]. Thus, it's necessary to seek some efficacy and safety treatment methods. According to our previous pilot clinical study and animal studies, Chinese Herbal Medicine JCM-16021 has been shown to be a potential efficacy on the improvement of symptoms of irritable bowel syndrome[5-7]. Hence, a large scale clinical study was designed to further evaluate the efficacy and safety of JCM-16021 for diarrhea-predominant Irritable bowel syndrome (IBS-D) with traditional Chinese medicine (TCM) pattern of Liver Stagnation and Spleen deficiency (LSSD). This trial has been registered in the ClinicalTrial.gov with identification number NCT03457324. In the statistical analysis plan (SAP), we will provide a detailed description of the proposed data analysis.

2. Study design

2.1 Overview

The study is a multi-center, randomized, double-blind, placebo controlled clinical trial that assess the efficacy and safety of JCM-16021 for IBS-D patients with TCM pattern of LSSD. 392 eligible volunteers are enrolled in the study and are randomized with 1:1 ratio to receive either JCM-16021 or placebo. The whole study consists of two weeks run-in period, eight weeks treatment period, and eight weeks follow-up period after treatment. The study is blinded to patients, all assessors (including all Chinese Medicine Practitioners and research assistants), and statistician. In the study, the REDCap[8, 9] will be used to manage all data.

2.2 Sample size estimation

From our previous study, improvements in global IBS symptom were 52% in JCM-16021 group and 32% in western medicine group, respectively[7]. To verify whether the investigational drug is more effective than placebo (the difference of improvement rate of symptoms is 15%), the StudySize2.0 software was used to calculate the sample size[10]. Assuming the improvement in the treatment group is 52%, p value <0.05 and statistical power is 80%, 166 patients per arm are needed. Further assuming a 15% dropout rate, a total of 392 patients (196 per arm) will be enrolled to ensure statistically significant results.

2.3 Definitions of the outcomes

2.3.1 Primary outcome

The primary outcome is defined as the global symptom improvement rate which is the percentage of patients with 4-6 score on Global Assessment of Improvement (GAI) score[11] at week 10. This global symptom improvement score will be also measured at week 6 and at week 18.

2.3.2 Secondary outcomes

a. IBS symptoms

***Pain Responder Rate In Daily Worst Abdominal Pain Scores*:** Pain responder rate is defined as the percentage of patients who are pain responder. Pain responder is defined as that participant who meets the daily pain response criteria for at least 50% of the days with diary entries during the observational period of interest. Daily pain response is defined as the ≥ 30% decrease in the worst abdominal pain scores in the past 24 hours compared to baseline (average of daily worst abdominal pain the 2-week prior to randomization)[12].

***Stool consistency Responder Rate In Daily Stool Consistency Scores*:** Stool consistency responder rate is defined as the percentage of patients who are stool consistency responder. Stool consistency responder is defined as that participant who meets daily stool consistency response criterion (ie, score of 1, 2, 3, or 4 or absence of bowel movement if accompanied by ≥30% decrease in worst abdominal pain scores compared to baseline pain) for at least 50% of days with diary entries during the observational period of interest[12].

Improvement Rate and Efficacy Rate: Improvement rate is defined as the percentage of patients in excellent, effective and helpful on single IBS symptom. Efficacy rate is defined as the percentage of patients with clinical remission and symptom improvement on comprehensive efficacy judgement of cardinal symptoms. The standards of efficacy assessment of single symptom are follows: a) Excellent: symptoms disappearing; b) Effective: symptom score decreased ⩾2 points; c) Helpful: symptom score decreased 1 point; d) Invalid: no change in symptom score. Comprehensive efficacy judgement standard of cardinal symptoms is calculated by "(total scoring of prior treatment – total scoring of post treatment) / total scoring of prior treatment x 100%". Clinical remission is defined as symptom disappearing; Symptom improvement is ≥80%; Helpful is between 50% and 80%; Invalid is <50%.[13]

IBS symptoms, including abdominal pain (with scores from 0 to 10 representing none to the most severe), diarrhea frequency and stool consistency will be recorded in Case Report Forms (CRFs). Investigators will grade each symptom by Cardinal symptoms evaluation quantitation scale (None=0, Mild=1, Moderate=2, Severe=3) shown in

Appendix 1, Table S1. The scores on IBS symptoms are measured at week 2, week 6, week 10 and week 18.

b. TCM pattern

Effective rate on TCM pattern is defined as the percentage of patients with clinical remission, excellence, and effective. The standards of efficacy assessment of TCM pattern are follows: Clinical remission: clinical symptoms and signs disappear or basically disappear, total scoring declining ⩾95%; Excellence: clinical symptoms and signs are significantly improved, total scoring declining ⩾ 70%; Effective: clinical symptoms and signs are improved, total scoring declining ⩾ 30%; Invalid: clinical symptoms and signs are without obvious improvement or even with exacerbation, total scoring declining < 30%.[13] Five typical symptoms for LSSD are assessed to evaluate the changes of TCM Pattern. These symptoms involve i) abdominal distension, ii) borborygmus and flatus, iii) distension and fullness in chest and hypochondrium, iv) frequent sighing, poor appetite, and v) mental depression or irritability. Investigators will grade the TCM pattern scale (None=0, Mild=1, Moderate=2, Severe=3) as shown in Appendix 1, Table S2. The effective rate on TCM pattern will be evaluated with following calculation formula (nimodipine method): [(total score of prior treatment – total score of post treatment) / total score of prior treatment]×100%[17]. The scores on TCM pattern are measured at at week 2, week 6, week 10 and week 18.

c. Irritable Bowel Syndrome-Quality of Life Score

Standard scores on Irritable Bowel Syndrome-Quality of Life (IBS-QoL) are measured at week 2, week 6, week 10 and week 18. The change of IBS-QoL score before and after the medication will be used to evaluate the improvement of quality of life[14].

d. Irritable Bowel Syndromes-Symptom Severity Score

The change of Irritable Bowel Syndromes-Symptom Severity (IBS-SSS) score before and after the medication will be used to evaluate the improvement of symptom severity[15]. Scores on IBS-SSS are measured at week 2, week 6, week 10 and week 18.

2.3.3 Safety outcome

The occurrence of adverse events related to Investigational medicinal products (IMP) (AEs) (including new incurrence symptoms or diseases, abnormal vital signs, clinically significant abnormal laboratory examination) or serious adverse events (SAEs) are used for safety evaluation.

3. Statistical Analysis

3.1 Analysis principles

- All treatment groups comparisons except for the primary outcome will be performed at a two-sided level of 0.05.

- Missing values of the primary outcome will be imputed by the last-observation-carried forward (LOCF) method.
- Both per-protocol analysis (PP) and intention-to treat (ITT) analysis will be used to conduct the efficacy analysis.
- Intention-to-treat principle will be used to deal with the non-compliance.
- All analysis will be exploratory analysis except for the one for the primary outcome.
- Subgroup analyses will be carried out irrespective of whether there is a significant treatment effect on the primary outcome.
- For the repeatedly measured outcomes, the difference with the baseline data (follow-up minus the baseline) will be analyzed and the baseline data will be included in the model as covariables.
- A Cochran-Mantel-Haenszel (CMH) test stratified by center will be used to test the difference between treatment groups.
- Analyses will be conducted primarily using SAS software (version 9.4, SAS Institute, Cary, North Carolina, USA). Figures will be plotted using R packages.

3.2 Trial profile

The flow chart of inclusion and follow-up will be displayed in a standard Consolidated Standards of Reporting Trials (CONSORT) diagram (Appendix 2, Figure S1). The report will include the number of patients included, withdrawn, lost to follow-up, the number who received the allocated treatment and were analyzed.

3.3 Patients' characteristics and baseline comparisons

Description and statistical inference of the baseline characteristics will be conducted by treatment groups (Appendix 1, Table S3). Discrete variables will be summarized by frequencies and percentages. Percentages will be calculated according to the number of patients for whom data are available. Continuous variables will be summarized by the use of standard meansures of central tendency and dispersion, or mean and standard deviation [Mean±SD].

3.4 Primary outcome

The primary outcome is the global symptom improvement rate which is the percentage of patients with 4-6 score on Global Assessment of Improvement (GAI) score at week 10. The results will be summarized using frequency (percentage) (Appendix 1, Table S4). Comparison of the global symptom improvement rate between the intervention group and placebo group will be analyzed by using chi-square test or CMH chi-square test when considering multicenter character.

3.5 Secondary outcome

Pain Responder Rate In Daily Worst Abdominal Pain Scores**:** Pain responder will be summarized using frequency (Appendix 1, Table S4). Comparison of pain responder rate between the intervention group and placebo group will be analyzed by using chi-square test or CMH chi-square test when considering multicenter character.

Stool consistency Responder Rate In Daily Stool Consistency Scores: Stool consistency responder will be summarized using frequency (Appendix 1, Table S4). Comparison of stool consistency responder rate between the intervention group and placebo group will be analyzed by using chi-square test or CMH chi-square test when considering multicenter character.

Improvement Rate and Efficacy Rate: Improvement rate on single IBS symptom and efficacy rate on comprehensive efficacy judgement of cardinal symptoms will be summarized using percentage (Appendix 1, Table S4). Comparison of improvement rate and efficacy rate between the intervention group and placebo group will be analyzed by using chi-square test or CMH chi-square test when considering multicenter character.

TCM pattern: Effective rate on TCM pattern will be summarized using percentage (Appendix 1, Table S4). Comparison of effective rate of TCM pattern between the intervention group and placebo group will be analyzed by using chi-square test or CMH chi-square test when considering multicenter character.

Irritable Bowel Syndrome-Quality of Life: The standard scores of IBS-QoL in each visit will be reported as mean ± standard deviation (M±SD) (Appendix 1, Table S4). Intra-group comparisons between baseline and each visit will be conducted by using paired t-test or Wilcoxon signed rank test. Comparisons between two groups will be conducted by using an analysis of variance (ANOVA), with other confounding factors like multicenter character conducting the covariate analysis. Statistical analysis for the data which do not meet above conditions (e.g. non-normal) will be conducted with the use of non-parametric test.

Irritable Bowel Syndromes-Symptom Severity Score: The score of IBS-SSS in each visit will be reported as mean ± standard deviation (M±SD) (Table S4). Intra-group comparisons between baseline and each visit will be conducted by using paired t-test or Wilcoxon signed rank test. Comparisons between two groups will be conducted by using an analysis of variance (ANOVA), with other confounding factors like multicenter character conducting the covariate analysis. Statistical analysis for the data which do not meet above conditions (e.g. non-normal) will be conducted with the use of non-parametric test.

3.6 Adverse events

Adverse events will be summarized using frequency (percentage) (Appendix 1, Table S5). Comparisons of incidence rate of AEs between intervention group and control group will be conducted with the use of chi-square test. Investigators need to list and describe the AEs happened in this trial. If the data do not conform to chi-square test (data include 0, or theoretical frequency is below 5), Fisher's exact test will be used. Population for safety analysis refers to all participants who enter the trial, receive medication at least one time and have suitable follow-up data for safety analysis. All safety data including AEs and laboratory results from participants will be assessed.

3.7 Patients' drop out

During the study, patients may drop out. The intention-to-treat principle will be used to deal with patients who dropped out. ITT population refers to all participants who go through randomization, enter double-blind treatment period, and receive medication at least one time. Participants who are lost to follow up or withdrawn from the study after randomization will be treated as dropout. The patients' drop-out will be descriptively summarized.

3.8 Subgroup analysis

The subgroup analysis will conducted for the improvement rate in GAI at week 10 as below:

-Grouped by 80%-120% / 70%-80% / less than 70% of medication consumption

-Grouped by age

-Grouped by gender

-Grouped by study centers

3.9 Sensitivity analysis

To test the robustness of the primary outcome analysis, the per-protocol (PP) principle analysis will be performed. PP population refers to all participants who complete relative observation according to protocol requirement and are confirmed to meet following conditions:① Compliance is between 80% and 120% (The compliance is calculated by "actual medication consumption / expected medication consumption × 100%".); ②No taking prohibited medications during the process of trial; ③Meeting inclusion criteria and not fitting any exclusion items; ④Completing all planned visits and necessary items of CRF.

3.10 Trial status

The trail has completed the process of recruitment. Before the data analysis, the

database will be cleaned and checked. The database will be locked once the SAP is finalized by principle investigator and statistician.

3.11 Tables and figures for the main results paper

The proposed tables and figures for the main results are presented in Appendix 1 and 2.
Table S1 will report cardinal symptoms evaluation quantitation scale.
Table S2 will report the grade of TCM pattern degree.
Table S3 will report the baseline characteristics of participants in the intervention and control group.
Table S4 will report the results of the primary outcome and secondary outcomes.
Table S5 will report the adverse events related to IMP.

References:

1. Ford AC, Lacy BE, Talley NJ: Irritable Bowel Syndrome. The New England journal of medicine 2017, 376(26):2566-2578.
2. Mearin F, Lacy BE, Chang L, Chey WD, Lembo AJ, Simren M, Spiller R. Bowel Disorders. Gastroenterology 2016.
3. Lacy BE, Pimentel M, Brenner DM, et al. ACG Clinical Guideline: Management of Irritable Bowel Syndrome. Am J Gastroenterol. 2021;116(1):17-44.
4. Sperber AD, Bangdiwala SI, Drossman DA, et al. Worldwide Prevalence and Burden of Functional Gastrointestinal Disorders, Results of Rome Foundation Global Study. Gastroenterology. 2021;160(1):99-114.e3.
5. Bian ZX, Zhang M, Han QB, Xu HX, Sung JJ. Analgesic effects of JCM-16021 on neonatal maternal separation-induced visceral pain in rats. World J Gastroenterol. 2010;16:7.
6. Qin HY, Xiao HT, Leung FP, Yang ZJ, Wu JC, Sung JJ, et al. JCM-16021, a Chinese Herbal Formula, Attenuated Visceral Hyperalgesia in TNBS-Induced Postinflammatory Irritable Bowel Syndrome through Reducing Colonic EC Cell Hyperplasia and Serotonin Availability in Rats. Evid Based Complement Alternat Med. 2012:239638.
7. Leung WK, Wu J, Bian ZX, Suen B, Ziea E, Yim C, et al. A pilot study comparing traditional Chinese herbal medicine or hyoscine on treatment of irritable bowel syndrome. J Gastroenterol Hepatol. 2007;22.
8. Harris PA, Taylor R, Thielke R, Payne J, Gonzalez N, Conde JG. Research electronic data capture (REDCap)--a metadata-driven methodology and workflow process for providing translational research informatics support. J Biomed Inform. 2009;42(2):377-381.

9. Harris PA, Taylor R, Minor BL, et al. The REDCap consortium: Building an international community of software platform partners. J Biomed Inform. 2019;95:103208.
10. Corazziari E, Bytzer P, Delvaux M, Holtmann G, Malagelada JR, Morris J, Muller-Lissner S, Spiller RC, Tack J, Whorwell PJ. Clinical trial guidelines for pharmacological treatment of irritable bowel syndrome. Aliment Pharmacol Ther. 2003;18:6.
11. Gordon S, Ameen V, Bagby B, Shahan B, Jhingran P, Carter E. Validation of irritable bowel syndrome Global Improvement Scale: an integrated symptom end point for assessing treatment efficacy. Dig Dis Sci. 2003;48:7.
12. Lembo AJ, Lacy BE, Zuckerman MJ, et al. Eluxadoline for Irritable Bowel Syndrome with Diarrhea. N Engl J Med. 2016;374:3.
13. Zhen XY. Guiding Principles for Clinical Study of New Chinese Medicines. The guiding principles of clinical research on the treatment of diarrhea with new Chinese medicine. Beijing: China Medical Science Press; 2002. p.139-143.
14. Patrick DL, Drossman DA, Frederick IO, DiCesare J, Puder KL. Quality of life in persons with irritable bowel syndrome: development and validation of a new measure. Dig Dis Sci. 1998;43:2.
15. Francis CY, Morris J, Whorwell PJ. The irritable bowel severity scoring system: a simple method of monitoring irritable bowel syndrome and its progress. Aliment Pharmacol Ther. 1997;11:2.

Appendix 1: Proposed format of data tables in the publication of the main results

Table S1: Cardinal symptoms evaluation quantitation scale

Symptom	Grading	Illness degree
Abdominal pain (0-10 numeric pain intensity scale)	None	No abdominal pain or discomfort
	Mild	1-3 indicate mild pain
	Moderate	4-6 indicate moderate pain
	Severe	7-10 indicate severe pain
Frequency of diarrhea (times/day)	None	No symptoms
	Mild	3-4 times/day
	Moderate	5-6 times/day
	Severe	⩾7 times/day
Stool consistency	None	Like sausage but smooth and soft
	Mild	Soft blobs with clear cut edges
	Moderate	Fluffy pieces with ragged edges
	Severe	Watery with no solid pieces

Table S2: The grade of TCM pattern degree

Symptom	Grading	Illness degree
Abdominal distension	None	No symptom
	Mild	Distension after meal, spontaneous remission in half an hour
	Moderate	Relatively heavier distension but bearable
	Severe	Persistent abdominal distention
Borborygmus, flatus	None	No symptom
	Mild	Occasional borborygmus and flatue
	Moderate	Frequent borborygmus and flatue
	Severe	Persistent borborygmus and flatue
Fullness in chest and hypochondrium	None	No symptom
	Mild	Occasional happens
	Moderate	Generally happens in emotional fluctuation, obvious fullness
	Severe	Persistent and intolerable fullness, need medication to release
Preference for sighing	None	No symptom
	Mild	Occasional happens
	Moderate	Generally happens in emotional fluctuation
	Severe	Frequently happens
Anorexia	None	No symptom
	Mild	Poor appetite
	Moderate	Appetite reduced less than 1/3 comparing with regular
	Severe	Appetite reduced more than 1/2 comparing with regular
Depression or irritability	None	No symptom
	Mild	Occasional depression or irritability
	Moderate	Easy-happening depression or irritability
	Severe	Frequent depression, or uncontrolled irritability

TCM: Traditional Chinese Medicine

Table S3. Baseline characteristics of participants in the intervention and control group

Characteristics	JCM-16021 Group (N=XXX)	Placebo Group (N=XXX)
Mean of age(SD), y	XX.XX±XX.XX	XX.XX±XX.XX
Sex, n(%)		
Female	XXX(XX.X%)	XXX(XX.X%)
Male	XXX(XX.X%)	XXX(XX.X%)
Mean body mass index(SD), Kg/m2	XX.XX(XX.XX)	XX.XX(XX.XX)
Mean heart rate(SD), beats/min	XX.XX(XX.XX)	XX.XX(XX.XX)
Mean systolic blood pressure (SD), mm Hg	XX.XX(XX.XX)	XX.XX(XX.XX)
Mean diastolic blood pressure (SD), mm Hg	XX.XX(XX.XX)	XX.XX(XX.XX)
History of IBS symptoms		
Mean duration of abdominal pain (SD), years	XX.XX(XX.XX)	XX.XX(XX.XX)
Mean duration of abdominal discomfort(SD), years	XX.XX(XX.XX)	XX.XX(XX.XX)
Mean duration of diarrhea(SD), years	XX.XX(XX.XX)	XX.XX(XX.XX)
Degree of IBS symptoms		
Mean score of abdominal pain (SD)	XX.XX(XX.XX)	XX.XX(XX.XX)
Mean score of stool consistency(SD)	XX.XX(XX.XX)	XX.XX(XX.XX)
Mean frequency of diarrhea(SD)	XX.XX(XX.XX)	XX.XX(XX.XX)
Degree of TCM* pattern		
Mean score of abdominal distention(SD)	XX.XX(XX.XX)	XX.XX(XX.XX)
Mean score of borborygmus, flatus (SD)	XX.XX(XX.XX)	XX.XX(XX.XX)
Mean score of fullness in chest and hypochondrium (SD)	XX.XX(XX.XX)	XX.XX(XX.XX)
Mean score of preference for sighing (SD)	XX.XX(XX.XX)	XX.XX(XX.XX)
Mean score of anorexia (SD)	XX.XX(XX.XX)	XX.XX(XX.XX)
Mean score of depression or irritability (SD)	XX.XX(XX.XX)	XX.XX(XX.XX)
Mean IBS-QoL score(SD)	XX.XX(XX.XX)	XX.XX(XX.XX)
Mean IBS-SSS score(SD)	XX.XX(XX.XX)	XX.XX(XX.XX)

TCM: Traditional Chinese Medicine; IBS: Irritable bowel syndrome; IBS-QoL: Irritable Bowel Syndrome-Quality of Life; IBS-SSS: Irritable Bowel Syndromes-Symptom Severity Score

Table S4. Results of the primary outcome and secondary outcomes

Outcome	ITT population		Between-Group Difference (95% CI)	P value
	JCM-16021 Group (N=XXX)	Placebo Group (N=XXX)		
Primary outcome				
Improvement rate in GAI (n (%))				
At week 10	XXX(XX.X%)	XXX(XX.X%)	XX(XX.XX to XX.XX)	
Secondary outcome				
Pain responder rate in Daily Worst Abdominal Pain Scores (n (%))				
At week 6	XXX(XX.X%)	XXX(XX.X%)	XX(XX.XX to XX.XX)	
At week 10	XXX(XX.X%)	XXX(XX.X%)	XX(XX.XX to XX.XX)	
At week 18	XXX(XX.X%)	XXX(XX.X%)	XX(XX.XX to XX.XX)	
Stool consistency responder rate in Daily Stool Consistency Scores (n (%))				
At week 6	XXX(XX.X%)	XXX(XX.X%)	XX(XX.XX to XX.XX)	
At week 10	XXX(XX.X%)	XXX(XX.X%)	XX(XX.XX to XX.XX)	
At week 18	XXX(XX.X%)	XXX(XX.X%)	XX(XX.XX to XX.XX)	
Improvement rate in pain degree (n (%))				
At week 6	XXX(XX.X%)	XXX(XX.X%)	XX(XX.XX to XX.XX)	
At week 10	XXX(XX.X%)	XXX(XX.X%)	XX(XX.XX to XX.XX)	
At week 18	XXX(XX.X%)	XXX(XX.X%)	XX(XX.XX to	

			XX.XX)
Improvement rate on stool consistency degree (n (%))			
At week 6	XXX(XX.X%)	XXX(XX.X%)	XX(XX.XX to XX.XX)
At week 10	XXX(XX.X%)	XXX(XX.X%)	XX(XX.XX to XX.XX)
At week 18	XXX(XX.X%)	XXX(XX.X%)	XX(XX.XX to XX.XX)
Improvement rate in diarrhea degree (n (%))			
At week 6	XXX(XX.X%)	XXX(XX.X%)	XX(XX.XX to XX.XX)
At week 10	XXX(XX.X%)	XXX(XX.X%)	XX(XX.XX to XX.XX)
At week 18	XXX(XX.X%)	XXX(XX.X%)	XX(XX.XX to XX.XX)
Efficacy rate in comprehensive efficacy judgement of cardinal symptoms (n (%))			
At week 6	XXX(XX.X%)	XXX(XX.X%)	XX(XX.XX to XX.XX)
At week 10	XXX(XX.X%)	XXX(XX.X%)	XX(XX.XX to XX.XX)
At week 18	XXX(XX.X%)	XXX(XX.X%)	XX(XX.XX to XX.XX)
Effective rate in TCM pattern (n (%))			
At week 6	XXX(XX.X%)	XXX(XX.X%)	XX(XX.XX to XX.XX)
At week 10	XXX(XX.X%)	XXX(XX.X%)	XX(XX.XX to XX.XX)
At week 18	XXX(XX.X%)	XXX(XX.X%)	XX(XX.XX to XX.XX)
Change in IBS-QoL score(Mean±SD)			

At week 6	XX.XX± XX.XX	XX.XX± XX.XX	XX(XX.XX to XX.XX)
At week 10	XX.XX± XX.XX	XX.XX± XX.XX	XX(XX.XX to XX.XX)
At week 18	XX.XX± XX.XX	XX.XX± XX.XX	XX(XX.XX to XX.XX)
Change in IBS-SSS score(Mean±SD)			
At week 6	XX.XX± XX.XX	XX.XX± XX.XX	XX(XX.XX to XX.XX)
At week 8	XX.XX± XX.XX	XX.XX± XX.XX	XX(XX.XX to XX.XX)
At week 18	XX.XX± XX.XX	XX.XX± XX.XX	XX(XX.XX to XX.XX)

GAI: Global Assessment of Improvement; TCM: Traditional Chinese Medicine; IBS-QoL: Irritable Bowel Syndrome-Quality of Life; IBS-SSS: Irritable Bowel Syndromes-Symptom Severity Score.

Table S5. Adverse events related to IMP.

Adverse Events	JCM-16021 Group (N=196)		Placebo Group(N=196)		P value
	Participants, n (%)	Events, n	Participants, n (%)	Events, n	
Total					
Level 1*					
XX					
Level 2*					
XX					
Level 3*					
XX					
Level 4*					
XX					
Level 5*					
XX					

IMP: Investigational medicinal products

*The severity of adverse events are graded according to the Common Terminology Criteria for Adverse Events Version 4.0.

Appendix 2: Figure for the main results paper.

Figure S1. Flowchart of inclusion and follow-up.

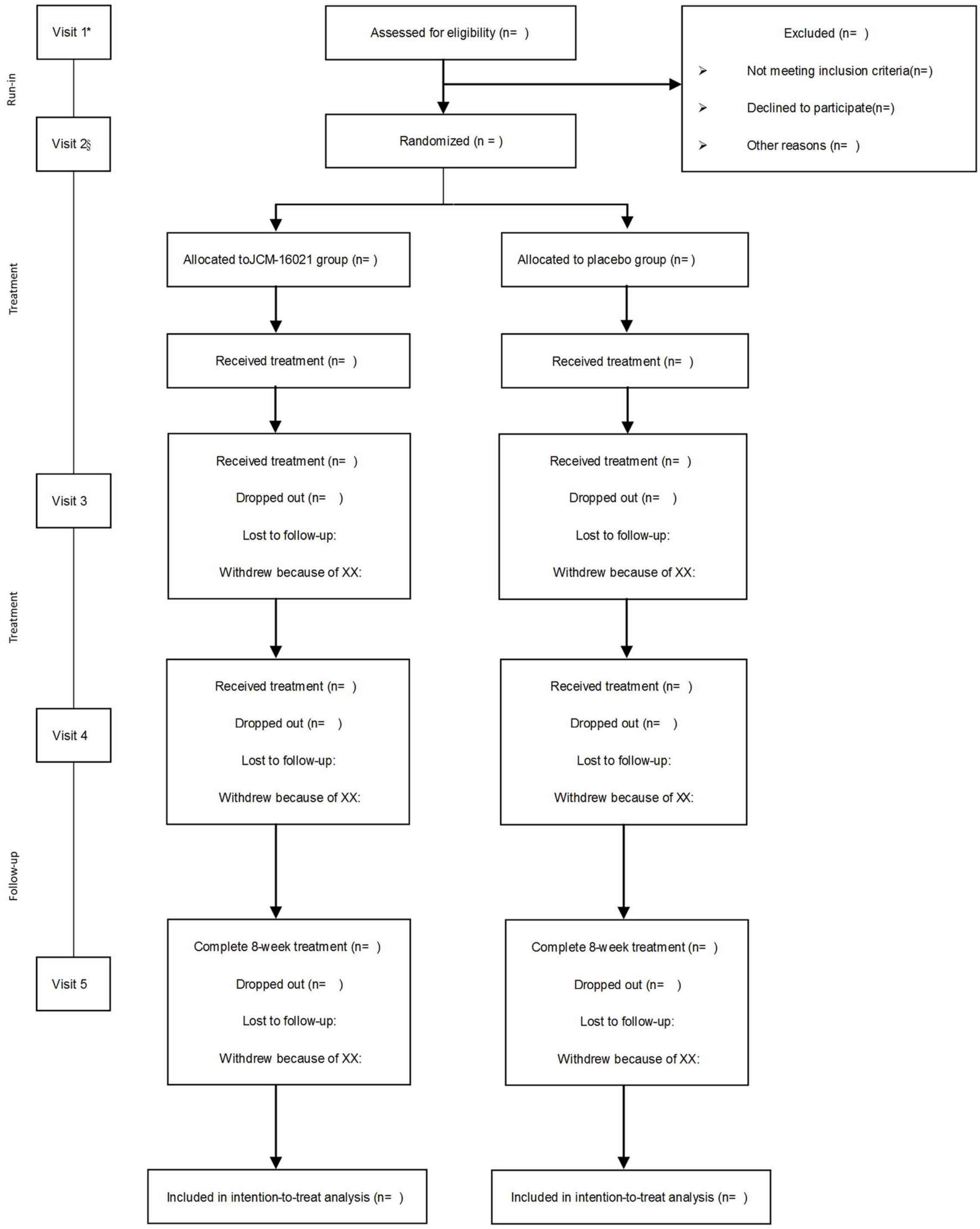

*Screening will be conducted at visit 1.

§Randomization will be conducted at visit 2. And the data collected at visit 2 will be used as baseline data.

Appendix 3: Statement of contribution of the authors

ZXB and JW are the principal investigators of the trial. ZXB and JW manage the progress of the trial. JC, LLDZ, KLC, WCL participate to design the protocol. YZ drafts the manuscript of the protocol. JC also helps to develop the protocol and monitor the study. So JC and YZ contribute equally and could be considered as co-first authors. CWC revises the manuscript of the protocol. YZ, XZ, CWC, PYL, XYW, and MK contribute to enroll the participants in the site of Hong Kong Baptist University clinics. KLC, PKC, CWL, and JC manage and promote the recruitment of participants in the site of the Chinese University of Hong Kong clinics. YZ drafts the manuscript of the SAP. PHC participates in writing the SAP. The SAP is prepared without knowledge of the data. ZXB finalizes the manuscript of protocol and SAP. All the members of this study participate in critical reviews of the SAP and approve the all final manuscript.
