# Supplementary material for: Efficacy and safety of Chinese medicine JCM-16021 for diarrhea-predominant irritable bowel syndrome: study protocol for a multi-center, randomized, double-blind, placebo controlled clinical trial
Source: Chin Med. 2021 Nov 13;16:117. doi: 10.1186/s13020-021-00530-2 (PMC8590321; doi:10.1186/s13020-021-00530-2)
Supplement: Supplementary file 4 — Additional file 4. Informed Consent Form. [file 13020_2021_530_MOESM4_ESM.pdf]

HONG KONG BAPTIST UNIVERSITY
INFORMED CONSENT STATEMENT (version 7.0)

Efficacy and Safety of Chinese Medicine JCM-16021 for Diarrhea-predominant Irritable Bowel Syndrome: a Multicenter, Randomized, Double-blind, Placebo Controlled Clinical Trial

You are invited to participate in a research study to evaluate the efficacy and safety of a new Chinese medicine (JCM-16021) on diarrhea predominant irritable bowel syndrome (IBS-D) patients with Liver depression and Spleen deficiency (LDSD) syndrome.

Background
Irritable bowel syndrome (IBS) is a functional gastrointestinal disorder which hallmark is abdominal pain or discomfort associated with a change in the consistency or frequency of stools. The prevalence of IBS is up to 20% in some developed countries. In Hong Kong, the prevalence among Chinese was 6.6%, while IBS symptoms like urgency straining, feeling of incomplete defecation, mucus in stool and abdominal distension was 11.6%, 41.7%, 11.1% and 37.7%, respectively. This disease is one of the most common conditions leading to seek of medical care.

JCM-16021 Granules is a new Chinese herbal medicine (CHM) drug with ingredients of Rhizoma Atractylodis Macrocephalae (Baizhu), Radix Paeoniae Lactiflorae (Baishao), Cortex Magnoliae Officinalis (Houpo), Semen coicis Lachryma-jobi (Yiyiren), Polygonaceae (Huotanmu), Fructus Terminaliae Chebulae (Hezi), and Rhizoma Corydalis Yanhusuo (Yanhusuo) tailor-made for treating diarrhea predominant IBS patients with Liver depression and Spleen deficiency syndrome. The herbs of this formula has been used in clinical practice for a long period of time without serious reported adverse effects. Besides, from raw materials purchased, to production process and packing, the quality control is strictly compliance with international GMP (Good Manufacturing Practice) standard.

Study Aims
We have conducted a series of basic and clinical researches on developing JCM-16021 Granules from 2004; however, a large scale clinical trial to evaluate its efficacy and safety is crucial. We believe this study can provide consolidated evidence by comparing with placebo on the treatment of IBS-D.

According to previous basic research, JCM-16021 can effectively improve the composition of the intestinal flora in IBS-D animal models and regulate the amount of metabolites in the intestinal flora, and such changes in the microenvironment is related to the improvement of IBS-D intestinal symptoms. Therefore, the regulation of intestinal microflora is likely to be an important mechanism for the treatment of IBS-D by JCM-16021. To verify this point of view, we will perform qualitative and quantitative analysis of microorganisms and microbially derived metabolites in stool.

Project Title
Efficacy and Safety of Chinese Medicine JCM-16021 for Diarrhea-predominant Irritable Bowel Syndrome: A Multicenter, Randomized, Double-blind, Placebo Controlled Clinical Trial

Study Plan
It is a 18-week study, including 2-week run-in, 8-week treatment, 8-week follow up and 5 visits at week 0, 2, 6, 10 and 18. In total, 392 patients will be recruited in this study.

For ensure your suitability of participation, you need to complete a set of questionnaire, participants have to visit Chan & Hou Medical Laboratories Ltd. to provide urine, feces and blood samples. The routine blood test, urine for urinalysis, stool routine test, liver function test and renal function test will be carried out in Chan & Hou Medical Laboratories Ltd.. If the participant needs western medical diagnosis or colonoscopy to assess inclusion / exclusion criteria, investigators with relative qualifications from the Institute of Digestive Disease, Faculty of Medicine, The Chinese University of Hong Kong will assist. The relevant source documents will be kept at the Institute of Digestive Disease, Faculty of Medicine, The Chinese University of Hong Kong. If you fulfill the requirement, you will be invited to enter the second part of study (week 3-18). The medication you take is based on randomization sequence generated by computer in double blind manner. They are either (A) JCM-16021 Granules, or (B) Placebo. 3 visits will be arranged within the 8-wk treatment period and at the end of study at wk 6, 10 and 18 for providing medical consultation (routine blood test, urine for urinalysis, stool routine test, liver function test and renal function test at wk 10 will be carried out in Chan & Hou Medical Laboratories Ltd. in the same way as mentioned above) and recording your improvement and any adverse effect.

The participation in the study of changes in metabolism of the intestinal flora before and after administration is optional. We will examine the various biological samples provided by you on weeks 0 and 10 including blood, urine and stool for analysis, and to monitor changes in metabolic processes in your gut flora to provide the theoretical basis for subsequent phase III clinical trials. Samples (blood, urine and stool) will be submitted to the testing and analysis center of the School of Chinese Medicine, Hong Kong Baptist University for the study of metabolic changes in the intestinal flora before and after administration.

Risks and Emergency Medical Treatment

In the unlikely event of physical injury resulting from your participation in this research. Some participants may experience IBS-related symptoms during follow-ups. If necessary, participants can take medications or seek advice based on your own circumstances. Emergency medical treatment will be provided at no cost to you. Be certain that you immediately notify the researcher if you are injured. If you require additional medical treatment you will be responsible for the cost. No other compensation will be provided if you are injured in this research.

Urgent contact: Prof. Bian ZhaoXiang at 307, Jockey Club School of Chinese Medicine Building, 7 Baptist University Road, Kowloon Tong, 3411 2699.

Benefits

Research leads to many advances in diagnosis and treatment of illness. Taking part in this research may not only benefit to you individually, but also helps to find a treatment for IBS-D. Moreover, the experience of conducting this research definitely makes contribution on using Chinese herbal medicine in human beings and further researches about Traditional Chinese Medicine in the future.

Confidentiality

All information only for research used and no personal information would be released. However, the Ethics Committee, the regulatory authorities or the sponsor may be granted access to such information in accordance with required procedures.

Compensation and Insurance

You have no compensation and charges for participating of this study. After the completion of this study, you can still seek for medical treatment in our clinics at your own cost. The study is covered by professional liability insurance policy.

Early termination

Investigators have the right to require participants to quit the clinical trial or terminate their participation for any reasons related to participant's interests, including other comorbid illness and AEs, without regard to the subject's consent.

Contact

If you have questions at any time about the study or the procedures, you may contact the researcher, Prof. Bian ZhaoXiang at 307, Jockey Club School of Chinese Medicine Building, 7 Baptist University Road, Kowloon Tong, 3411 2699.

If you feel that you have not been treated according to the descriptions in this form, or your rights as a participant in this research have been violated during the course of this project, you may contact the Research Ethics Committee by email at hkbu_rec@hkbu.edu.hk (phone: 3411 7941).

Participation

Your participant in this study is voluntary; you have responsibility to understand the documentations and follow the clinical research process; you may decline to participate without penalty. If you decide to participate, you may withdraw from the study at any time without penalty and without loss of benefits to which you are otherwise entitled. If you withdraw from the study before data collection is completed your data will be returned to you or destroyed. Significant new findings developed during the course of the research, and which may be related to the subject's willingness to continue participation, will be provided to the subject.

Consent

I have read and understand the above information. I have received a copy of this form. I agree to participate in this study.

☐ I also agree<br>☐ I disagree	The collected samples (blood, urine, and faeces) will be submitted to the Testing and Analysis Center of the School of Chinese Medicine, Hong Kong Baptist University to study the changes in the metabolism of the intestinal flora before and after administration.

Signature of the Subject ____ Date ________________________

Signature of the Parent(s) / Guardian(s) ____________________________ Date ________________________

Signature of the Investigator ______________________________________ Date ________________________

香港浸會大學

仁朮腸樂顆粒治療腹瀉型腸易激綜合症有效性和安全性的多中心、隨機、雙盲、安慰劑對照臨床試驗

*同意聲明書（版本 **7.0**）*

閣下獲邀參加仁朮腸樂顆粒(JCM-16021)治療肝鬱脾虛泄瀉型腸易激綜合症(IBS-D)的療效及安全性評價。

資料背景
腸易激綜合症(IBS)是臨床常見的功能性胃腸病症，主要症狀為腹痛或腹部不適，以及相關的排便次數和習慣的改變。在一些發達國家，IBS的發病率甚至達到20%以上，香港的華人發病率約為6.6%，而急性排便費力、排便不暢、大便粘滯及腹部不適的比例分別為11.6%，41.7%，11.1%和37.7%。腸易激綜合症亦是導致患者求醫的最常見病症之一。

仁朮腸樂顆粒是針對肝鬱脾虛型IBS-D患者而研發的純中藥配方，成份包括白朮、白芍、厚朴，薏苡仁、火炭母、訶子和延胡索。該藥物的各種中藥已經在臨床上使用多年而未有出現嚴重的毒副作用。此外，本品從原料、生產到包裝均受到嚴格的品質監控，並符合國際優良藥品製造規範(GMP)的標準。

研究目的
從2004年開始，研究小組於研發仁朮腸樂顆粒的過程中進行一連串的實驗室及臨床研究，但是一個樣本量大的臨床研究來評估其療效及安全性仍然十分關鍵。我們希望可透過與安慰劑作比較，進一步證明仁朮腸樂顆粒治療IBS-D的療效和安全性。

根據前期基礎研究表明 JCM-16021 可有效改善 IBS-D 動物模型的腸道菌群的結構及調節腸腔內菌群代謝產物量，並且此微環境的變化與 IBS-D 腸道症狀的改善相關。所以，腸道微生態的調節很可能是一個重要的 JCM-16021 有效治療 IBS-D 的作用機制。為了驗證此觀點，我們將進行糞便中的微生物及其代謝產物的定性及定量分析研究。

計劃名稱
仁朮腸樂顆粒治療腹瀉型腸易激綜合症有效性和安全性的多中心、隨機、雙盲、安慰劑對照臨床試驗

研究計劃
本研究為期18個星期，當中包括2星期的篩選期、8星期的藥物治療、8星期的隨訪觀察，研究中包括有5次與研究人員者的會面(第0週、第2週、第6週、第10週及第18週)。整個研究將招募392位參加者。

為確定閣下適合參與研究，您需要完成一份問卷調查，並前往香港(CH)病理檢驗中心提交尿液、糞便和血液樣本。血常規、尿常規、大便常規、肝腎功能檢驗將在香港(CH)病理檢驗中心進行。若受試者需要西醫診斷或腸鏡檢查以評估納入/排除標準，將會由香港中文大學醫學院消化疾病研究所之研究者醫生負責協助。相關原始文件將保存於香港中文大學醫學院消化疾病研究所。若閣下符合所需條件，就會被邀請進入第二部份的研究(第3週至第18週)。閣下所接受之藥物組別是由雙盲式電腦隨機抽樣決定，分別為 (A)中藥治療組(仁朮腸樂顆粒) 或(B)安慰劑組。在8週藥物治療期間及其後8週的觀察期，我們會為你安排複診(分別在第6週、第10週及第18週進行，並於第10週在香港(CH)病理檢驗中心以上述相同方法進行血常規、尿常規、大便常規、肝腎功能檢驗)，由研究人員跟進病情變化、可能出現的副作用和服藥情況等事項。

閣下亦可自願參與有關用藥前後腸道內菌群代謝過程的變化研究，我們將研究閣下於第 0 週及第 10 週已提供的多種生物樣本 包括：血液，尿液及糞便作整合分析，並監測閣下腸腔內菌群代謝過程的變化，為後續的三期臨床實驗提供藥物治療的理論基礎。樣本(血液、尿液及糞便)將交由香港浸會大學中醫藥學院測試分析中心，研究用藥前後腸腔內菌群代謝過程的變化。

參加者簽署：

風險評估及緊急醫療措施

服食本中藥一般不會引起不良反應。部份參加者在觀察或隨訪期間或會出現 IBS 相關症狀，若有需要，參加者可按自身情況服用藥物或直接求診。若閣下的身體因參與這次研究而受到傷害，我們將負責相關的緊急醫療費用的開支。因此，如閣下的身體受到任何傷害，請立即通知研究人員。其他額外的醫療開支，則需由閣下自行支付。如果你在本研究中受傷，將不會提供任何其他補償。

緊急聯絡資料：卞兆祥教授　地址：九龍塘浸會大學道 7 號賽馬會中醫藥學院大樓 307 室　電話：3411 2699

研究效益

科學研究有助於醫學的發展，參與是次計劃除了可能幫助閣下改善 IBS 的症狀，同時本次研究的結果必定對研發中藥新藥治療腸易激綜合症有啟導性的作用。我們希望可透過科研，驗證中醫藥的療效，並且令中醫藥科研應用於改善人類的健康。

私隱保障

所有資料只作研究用途，任何個人資料均不會對外公佈。倫理委員會、有關的藥品監督管理部門或申辦者在工作需要時，按規定可以查閱參加試驗的受試者資料。

補償及保險安排

閣下參與是次的研究是沒有任何費用或額外酬勞的。在完成整個研究後，閣下可自費繼續在本校中醫診所繼續接受診治。本研究已投保專業責任保險，所有參加者均包括在保障範圍內。

中止試驗

研究人員有權以任何與臨床研究受試者的利益相關的理由，包括其它併發疾病或不良事件，或對於研究藥物的安全性或功效的評估，要求臨床研究受試者退出或中止臨床研究，而不用獲得受試者的同意。

聯絡資料

閣下對是次的研究有任何查詢，請即與研究小組人員提出，我們非常樂意為你作進一步解釋。

研究總負責人：卞兆祥教授　地址：九龍塘浸會大學道 7 號賽馬會中醫藥學院大樓 307 室　電話：3411 2699

如閣下對參與研究的相關權益有疑問，請致電聯絡倫理委員會代表：

香港浸會大學研究倫理委員會　電話：(852) 3411 7941　電郵：hkbu_rec@hkbu.edu.hk

參與條款

閣下是自願參與是次臨床研究，有責任了解相關文件資料及依從臨床研究的流程，並擁有隨時退出本研究的權利。閣下拒絕或提前退出參與本研究，是不會對你的醫療護理構成任何損失或懲罰的。如閣下在完成收集所需資料前決定退出本研究，所有已獲得的資料將會發還銷毀。研究的過程中若有顯著新發現，而新發現可能影響受試者考慮是否繼續參與，研究員會讓受試者選擇是否願意繼續參與。

病人同意書

本人已瞭解以上所有內容，持有同意聲明書副本，並自願同意參加本研究。

☐ 我也同意<br>☐ 我不同意	將已收集的樣本(血液、尿液及糞便)，交由香港浸會大學中醫藥學院測試分析中心，研究用藥前後腸腔內菌群代謝過程的變化。

參加者簽署 ______________________________　日期 ________________

監護人簽署 ______________________________　日期 ________________

研究人員簽署 ______________________________　日期 ________________
